# Structural, elastic, electronic, optical and vibrational properties of single-layered, bilayered and bulk molybdenite MoS_2_-2H

**DOI:** 10.1107/S1600576723002571

**Published:** 2023-04-25

**Authors:** Gianfranco Ulian, Giovanni Valdrè

**Affiliations:** a Biological, Geological and Environmental Sciences, University of Bologna, P. Porta San Donato 1, Bologna, Emilia-Romagna 40127, Italy; b Interdisciplinary Centre of Biomineralogy, Crystallography and Biomaterials, University of Bologna, P. Porta San Donato 1, Bologna, Emilia-Romagna 40127, Italy; Australian Synchrotron, ANSTO, Australia

**Keywords:** molybdenite MoS_2_-2H, monolayered MoS_2_, bilayered MoS_2_, crystal chemistry, electronic properties, dielectric properties

## Abstract

In recent years, transition metal dichalcogenides have received great attention since they can be prepared as two-dimensional semiconductors, presenting heterodesmic structures incorporating strong in-plane covalent bonds and weak out-of-plane interactions, with an easy cleavage/exfoliation in single or multiple layers. In this context, molybdenite, the mineralogical name of molybdenum disulfide, MoS_2_, has drawn much attention because of its very promising physical properties for optoelectronic applications, in particular a band gap that can be tailored with the material’s thickness, optical absorption in the visible region and strong light–matter interactions due to the planar exciton confinement effect. Despite this wide interest and the numerous experimental and theoretical articles in the literature, these report on just one or two specific features of bulk and layered MoS_2_ and sometimes provide conflicting results. For these reasons, presented here is a thorough theoretical analysis of the different aspects of bulk, monolayer and bilayer MoS_2_ within the density functional theory (DFT) framework and with the DFT-D3 correction to account for long-range interactions. The crystal chemistry, stiffness, and electronic, dielectric/optical and phonon properties of single-layered, bilayered and bulk molybdenite have been investigated, to obtain a consistent and detailed set of data and to assess the variations and cross correlation from the bulk to single- and double-layer units. The simulations show the indirect–direct transition of the band gap (*K*–*K*′ in the first Brillouin zone) from the bulk to the single-layer structure, which however reverts to an indirect transition when a bilayer is considered. In general, the optical properties are in good agreement with previous experimental measurements using spectroscopic ellipsometry and reflectivity, and with preliminary theoretical simulations.

## Abstract 2

An extensive density functional theory analysis is reported of the crystal-chemical properties, electronic band structure, and optical (absorption and electron energy-loss spectra) and phonon properties of bulk and mono- and bilayer molybdenite, a transition metal dichalcogenide with important applications in semiconductor technology. This mineral is characterized by virtually infinite bidimensional layers of MoS_2_ (with covalent Mo—S bonds) held together by weak dispersive forces, explaining the easy cleavage of the (001) surfaces.

## 1. Introduction

**Figure 1:**
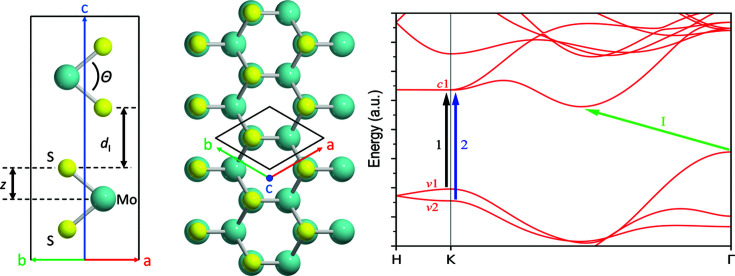
(Left and middle) Ball-and-stick models of bulk molybdenite MoS_2_-2H viewed along the (left) [110] and (middle) [001] directions. The black lines represent the hexagonal unit cell of the mineral. The S—Mo—S bond angle Θ, the interlayer distance *d*_I_ and the *z* parameter (*i.e.* the atomic shift of sulfur with respect to molybdenum atoms, scaling with the length of the *c* axis) are reported. (Right) The electronic band structure of MoS_2_-2H, highlighting the indirect band gap (I, green line) and the excitonic *K*–*K*′ (used in experimental work) transitions 1 (from valence band *v*1 to conduction band *c*1, black line) and 2 (*v*2 → *c*1, blue line).

Molybdenite (MoS_2_) is a sulfide mineral that crystallizes in the hexagonal crystal system (space group *P*6_3_/*mmc*) under standard pressure and temperature conditions. The unit cell of molybdenite is shown graphically in Fig. 1 ▸, presenting two formula units (*Z* = 2) of molybdenum disulfide, each one forming a layer where the atoms are linked by mixed covalent/ionic bonds. The molybdenum atom is sixfold coordinated, with a trigonal prism arrangement of the S^2−^ ions. These layers are extended indefinitely along the *a* and *b* axes and stacked along the *c*-axis direction. The whole structure is held along the [001] direction by weak long-range (van der Waals) interactions. Because the crystal structure is made up of stacked layers, molybdenite is subject to polytypism, and the structural model in Fig. 1 ▸ represents the MoS_2_-2H polymorph stable under standard conditions of pressure and temperature (1 atm, 298.15 K; 1 atm = 101 325 Pa).

Molybdenite is a widely employed mineral phase in several and manifold geological, industrial and technological applications. It is the main ore mineral for the extraction of metallic molybdenum (Hess, 1924 ▸) and an element used in alloy steels, superalloys and pigments, just to cite a few examples (Kropschot, 2010 ▸). Bulk molybdenite can be used as a dry lubricant because of its very low friction coefficient (Martin *et al.*, 1993 ▸, 1994 ▸), even under high pressure (Wu *et al.*, 2022 ▸).

In addition, molybdenite was the first discovered semiconductor material in the early 20th century (Aminoff & Broome, 1935 ▸), and it has drawn much attention in the past decade for its use as a two-dimensional (2D) material in field-emission transistors (FETs) and other electronic devices, such as solar cells and light-emitting diodes (Radisavljevic *et al.*, 2011 ▸; Fontana *et al.*, 2013 ▸; Sebastian *et al.*, 2021 ▸; Wang *et al.*, 2021 ▸). Thanks to the weak interactions along the [001] direction, molybdenite is easily cleavable, and a monolayer of molybdenite was exfoliated by Joensen *et al.* (1986 ▸) using intercalated lithium. It was shown that a single layer of MoS_2_ has superior properties to graphene for the fabrication of FETs and optoelectronic devices because it presents a gap in the band structure (Radisavljevic *et al.*, 2011 ▸). This band gap is tunable by increasing or decreasing the number of layers of the material (Yang & Li, 2020 ▸), and even changes from an indirect gap of about 1.23 eV in bulk MoS_2_-2H (Kam & Parkinson, 1982 ▸) to a direct gap of about 1.8 eV in the monolayer (Kuc *et al.*, 2011 ▸). This indirect–direct band-gap transition is fundamental to enhancing light emission and optical absorption, and to inducing photoluminescence in the monolayer of molybdenite (Mak & Shan, 2016 ▸).

Several experimental and theoretical reports have investigated the structural, electronic and vibrational properties of bulk and layered molybdenite to provide a better understanding of the quantum confinement effects originating from ‘layering’ the mineral and to drive the development of MoS_2_-based materials for photonics and optoelectronic applications. However, most of them focused only on specific features, such as the band gap (Kuc *et al.*, 2011 ▸; Kam & Parkinson, 1982 ▸), the dielectric properties (Beal & Hughes, 1979 ▸; Kumar & Ahluwalia, 2012 ▸), the elasticity (Feldman, 1976 ▸; Peelaers & Van de Walle, 2014 ▸) and the vibrational properties (Ataca *et al.*, 2011 ▸; Funke *et al.*, 2016 ▸; Wieting & Verble, 1971 ▸). Some of these reports also showed conflicting results, such as a shift of some Raman modes when MoS_2_ is prepared in layers.

Because of the relevance of this material in the next generation of devices, and to provide a detailed and comprehensive cross-correlated analysis of the different properties of molybdenite and its layered derivatives, we have performed a series of *ab initio* simulations at the density functional theory (DFT) level of the structures of the MoS_2_-2H bulk, the monolayer (here labelled as MoS_2_-1L) and the bilayer (MoS_2_-2L). This choice is dictated by previous theoretical analyses (Kuc *et al.*, 2011 ▸), which highlighted the occurrence of the direct band gap only in the monolayer, with bilayer MoS_2_ behaving at the electronic level similarly to the bulk mineral. In the following, an analysis of the structures and elastic, electronic, dielectric and phonon properties is provided, cross-correlated, and discussed against previous results reported in the scientific literature. Most important in our work is that all the simulations were performed with a correction to include the van der Waals interactions in the physical treatment, a non-trivial choice when dealing with layered materials such as molybdenite, other transition metal dichalcogenides and phyllosilicates (Moro *et al.*, 2016 ▸).

## 2. Computational methods

The present simulations have been conducted by means of the *Vienna ab initio Software Package* (*VASP*) code (Kresse & Furthmüller, 1996 ▸; Kresse & Hafner, 1993 ▸) using the density functional PBE (Perdew *et al.*, 1996 ▸). The projector augmented-wave (PAW) basis set was expanded using a cut-off of 600 eV throughout the different calculations (Kresse & Joubert, 1999 ▸). The contribution of the weak van der Waals forces was included according to the DFT-D3 approach (Grimme *et al.*, 2011 ▸),

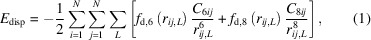

where the first two summations are over all *N* atoms in the cell and the third one is over the translations of the unit cell **L** = (*l*_1_, *l*_2_, *l*_3_). *C*_6*ij*_ and *C*_8*ij*_ are the dispersion coefficients of the atom pair *ij*, and *r*_*ij*,*L*_ is the distance between atom *i* in the reference cell (**L** = 0) and atom *j* in the cell **L**. The function *f*_d,*n*_ is the Becke–Jonson damping function, which is given by the expression

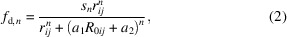

with *s*_6_ = 1 and *a*_1_, *a*_2_ and *s*_8_ as variable parameters depending on the geometry of the system.

Geometry optimization of the bulk (MoS_2_-2H) was conducted by means of an iterative procedure involving the successive minimization of the *a* lattice parameter and *c*/*a* ratio, starting from the experimentally refined unit cell (Schönfeld *et al.*, 1983 ▸). Within this procedure, the tolerance on forces was set at 10^−4^ eV Å^−1^. The molybdenite monolayer (MoS_2_-1L) and bilayer (MoS_2_-2L) structures were modelled as slabs containing just one and two structural layers, respectively, placed in a unit cell with the *a* axis equal to the refined *a* parameter of the bulk MoS_2_-2H. A vacuum region along the [001] direction of 20 and 30 Å thickness for MoS_2_-1L and MoS_2_-2L, respectively, was employed to avoid interaction between replicas of layers in neighbouring cells along the *c* axis. The sampling in the first Brillouin zone for the bulk mineral was conducted on a Monkhorst–Pack Γ-centred grid (Monkhorst & Pack, 1976 ▸) with 17 × 17 × 9 *k*-points for the geometry optimization procedure, whereas a finer mesh of 33 × 33 × 9 *k*-points was employed for the calculation of the electronic band structure, density of states and dielectric function. For the layered systems MoS_2_-1L and MoS_2_-2L, due to the increased *c* lattice parameter, the grids were reduced along the [001] direction to just one *k*-point, leading to 33 × 33 × 1 and 33 × 33 × 1 *k*-point meshes. For all the calculations, the threshold controlling the convergence on the total energy between two consecutive self-consistent field steps was set to 10^−8^ eV. Electronic properties (band structure and density of states) at the GW level of theory were obtained through generating maximally localized Wannier functions, which were calculated using the *Wannier90* code (Pizzi *et al.*, 2020 ▸). The frequency-dependent dielectric function was calculated with zero momentum transfer [**q** = 0; *q* = (4π/λ)sinθ, where θ is half the scattering angle and λ is the wavelength of the incident radiation] using both the independent particle approximation (without local field effects) (Gajdoš *et al.*, 2006 ▸) and the random phase approximation, the latter performed on top of a single-shot *G*_0_ *W*_0_ calculation (Shishkin & Kresse, 2006 ▸, 2007 ▸). The frequency-dependent dielectric function was then compared with known measures and models reported in the scientific literature. Phonon properties were calculated using the *Phonopy* package (Togo & Tanaka, 2015 ▸), calculating the Γ-point (**q** = 0) frequency and the phonon dispersion relations (**q** ≠ 0) using finite displacements and the modified Parlinski–Li–Kawazoe method (Parlinski *et al.*, 1997 ▸) on 3 × 3 × 3 and 3 × 3 × 1 supercells for the bulk and layered molybdenite models, respectively.

## 3. Results and discussions

### 3.1. Molybdenite structure

Geometry optimization is the first step necessary prior to any further analysis to assess the quality of the simulation approach. The structural results for the different molybdenite models (bulk, monolayer and bilayer) are reported in Table 1 ▸, alongside previous experimental and theoretical determinations. For the bulk mineral, our simulation at the PBE-D3 level of theory is correctly able to reproduce the structural features observed from the X-ray diffraction (XRD) refinements of Bronsema *et al.* (1986 ▸). In detail, we observed a small underestimation of the *a* and *c* lattice parameters of about −0.35 and −1.74%, respectively, which is an expected result at 0 K. The bond lengths are in good agreement with the XRD results, with a slight underestimation of about −1.88 and −0.46% for the S—S and Mo—S bonds, respectively. The structural data are also in good agreement with the experimental lattice parameters obtained via scanning tunnelling microscopy, *a* = 3.160 Å and *c* = 12.294 Å (Schönfeld *et al.*, 1983 ▸). Furthermore, our theoretical Mo—S and S—S bond lengths (2.399 and 3.149 Å, respectively, at the DFT-D3 level) are in good agreement with those measured experimentally (2.41 and 3.19 Å, respectively; Schönfeld *et al.*, 1983 ▸).

The binding energy between the MoS_2_ layers was calculated according to the following formula:

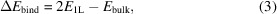

where *E*_bulk_ is the total energy of the bulk mineral and *E*_1L_ is the energy of a single layer (the factor 2 considers the presence of two layers in the bulk unit cell). Our theoretical value (Δ*E*_bind_ = 47 meV per atom = 0.456 J m^−2^) is in very good agreement with both the Δ*E*_bind_ = 0.52 (4) J m^−2^ obtained by Weiss & Phillips (1976 ▸) from the experimental specific surface energy of molybdenite and the binding energy value of 0.55 (13) J m^−2^ measured by Fang *et al.* (2020 ▸) using an *in situ* peeling-to-fracture method. In the latter study, the authors performed a theoretical simulation of the mechanical exfoliation of molybdenite with the *VASP* code using the optB86b-vdw kernel, which is a modified version of the vdW-DF approach. The results provided Δ*E*_bind_ = 0.422 J m^−2^, which is slightly lower than the value obtained from our simulations. We infer that this small deviation is due to the different computational settings employed by Fang *et al.* (2020 ▸), especially the different description of the van der Waals long-range interactions.

We also calculated the cohesive energy of bulk molybdenite using the formula

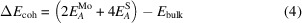

and of the layered structures using

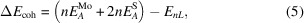

where 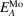 and 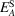 are the atomic energy of the isolated Mo and S atoms, respectively, *n* is the number of layers, and *E_nL_* is the energy of the layered system. The cohesive energy (5.438 eV per atom) is in good agreement with the experimental value of 5.18 eV per atom reported by Raybaud *et al.* (1997 ▸). These results are also in line with those of Bučko *et al.* (2013 ▸), who obtained Δ*E*_coh_ = 5.37 eV per atom. As explained by those authors, this slight overestimation is mainly related to a high contribution from the correction for the dispersive forces, since the sole PBE functional provided a cohesive energy closer to the experimental value. The Δ*E*_coh_ value that we obtained from the DFT-D3 approach seems to confirm this hypothesis.

Comparison with the results using the standard PBE functional (Bučko *et al.*, 2010 ▸) indicates that the inclusion of dispersive forces, at least via *a posteriori* corrections, plays a fundamental role in determining the physical–chemical properties of MoS_2_-2H and other layered materials, in agreement with previous statements on the topic (Cutini *et al.*, 2020 ▸; Ulian *et al.*, 2013 ▸, 2021 ▸). Indeed, the lack of an appropriate treatment results in an overly overestimated *c* lattice parameter and extremely low bulk modulus, as shown by Ataca *et al.* (2011 ▸). The uncorrected PW91 functional provided a good description of the structural information directly related to the MoS_2_ layers, *i.e.* the *a* lattice parameter, but the interlayer binding energy dropped to the negligible value of 3 meV per atom (−94%), resulting in a dramatic increase of the *c* lattice parameter (25% with respect to the PW91-D2 results). Hence, since most of a mineral’s properties (optical, electronic and so on) are strictly related to its structure, it is of the utmost importance to use a theoretical approach that considers all the relevant physical forces.

One of the first attempts to include dispersive forces in the physical description of layered structures (*e.g.* graphite and molybdenite) was provided by Rydberg *et al.* (2003 ▸), who included these kinds of forces in a nonlocal correlation density functional called vdW-DF, coded in the *PWscf* program. The structural results (lattice parameters and bulk modulus) provided were in reasonably good agreement with the available experimental data (Aksoy *et al.*, 2006 ▸; Bronsema *et al.*, 1986 ▸). Compared with the results of the present study, the vdW-DF approach provided a slightly overestimated unit-cell volume due to larger *a* and *c* parameters. More recently, in the work of Bučko *et al.* (2013 ▸), who employed the *VASP* code and a DFT-D2 correction for the dispersive forces, the calculated lattice parameters of the unit cell were *a* = 3.19 Å and *c* = 12.42 Å, which are slightly larger than those obtained in the present study, in particular for the *c* axis. This discrepancy is mainly related to the different correction for the dispersive forces, because the original D2 scheme of Grimme (2006 ▸) is more empirical than the D3, where the *C*_6_ parameters are adjusted for each atom according to the local chemical environment (charge density) in which it is located. Also, the 8 × 8 × 8 *k*-point mesh employed by Bučko *et al.* (2013 ▸) has fewer sampling points than the one adopted here (17 × 17 × 9), although the authors considered a very large kinetic energy cut-off (*i.e.* quality of the basis set) of 1500 eV.

Our simulation results for bulk MoS_2_-2H are also in good agreement with previous theoretical results at the DFT level reported by Ataca *et al.* (2011 ▸). In that work, the authors employed the *PWscf* package in *QuantumEspresso* (Giannozzi *et al.*, 2009 ▸), plane-wave basis sets with ultrasoft pseudo­potentials (Vanderbilt, 1990 ▸) and the PW91 density functional (Perdew *et al.*, 1992 ▸), corrected with the DFT-D2 scheme. In particular, the unit-cell lattice and internal geometry of bulk molybdenite obtained with our approach were within about 1% of those previously simulated, and no difference in the binding energy Δ*E*_bind_ was observed. We note that the formation energy from our simulation is about 6% more than that calculated at the PW91-D2 level, which may be due to the different choice of the density functional and basis sets.

Compared with the bulk mineral, the molybdenite monolayer (MoS_2_-1L) and bilayer (MoS_2_-2L) structures showed very small variations regarding their internal geometry, with differences not higher than 0.1%. This is a typical trend in layered minerals and in materials whose structural features do not vary significantly when simulated in layers (Ulian *et al.*, 2018 ▸). This can also be evinced from the cohesive energy of both the mono- and bilayer models, which were within 1% of that of bulk MoS_2_-2H. It is interesting that the difference between the cohesive energy of MoS_2_-1L and MoS_2_-2H corresponds to the binding energy per MoS_2_ unit, which is twice the Δ*E*_bind_ per atom. The same applies to the bilayer slab of molybdenite. Finally, and as expected, the binding energy between the two layers of the molybdenite bilayer structure is 22 meV per atom = 0.213 J m^−2^, which is half the Δ*E*_bind_ value of the bulk mineral because in the latter each MoS_2_ sheet interacts with one above and one below, whereas there is only a single layer-to-layer interaction in the bilayer model. The above observations and discussion are in line with the results reported by Ataca *et al.* (2011 ▸), who compared the structural properties of bulk and monolayer molybdenite. However, those authors did not consider a possible bilayer structure in their simulations.

### 3.2. Elasticity

To provide a further assessment of the quality of our simulation approach, we calculated the second-order elastic moduli of bulk molybdenite. The elasticity of bulk molyb­denite was obtained using a finite strain approach, calculating the stiffness moduli from the stress–strain relationship (Yu *et al.*, 2010 ▸; Nye, 1957 ▸). For a hexagonal structure there are five independent elastic moduli that, according to the 6 × 6 matrix notation of Voigt, can be expressed as

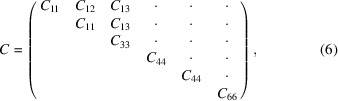

where *C*_66_ = (*C*_11_ − *C*_12_)/2 and the dots are null moduli. Three strain patterns were necessary to obtain the elastic constants and, for each of them, five equally spaced strain magnitudes between −0.015 and 0.015 were employed. For the calculation of the elastic moduli, the unit cell vectors **a** and **c** were oriented parallel to the *x* and *z* Cartesian axes, respectively, which is the standard crystallographic orientation of a hexagonal unit cell proposed by the Institute of Radio Engineering (Brainerd, 1949 ▸). The simulation results are presented in Table 2 ▸ and compared with previous experimental and theoretical data. Note the strong anisotropy between the *C*_11_ = 231.82 GPa and *C*_33_ = 63.47 GPa stiffness matrix components, which are related to elastic deformations along the *a* and *c* axes, respectively. As previously mentioned, this is a consequence of the different bonding scheme in molybdenite, where the Mo and S atoms are linked by strong covalent bonds and the MoS_2_ layers, stacked along the [001] direction, are held together by van der Waals interactions. A similar anisotropic behaviour has been observed for other layered phases as well (Gatta *et al.*, 2015 ▸; Ulian *et al.*, 2014 ▸).

These theoretical findings are in line with the approximate values calculated from different experimental measurements (neutron dispersion curves and compressibility data from XRD refinements) by Feldman (1976 ▸). Our results show an underestimation of about −2.6% for the *C*_11_ modulus and an overestimation of the *C*_13_ stiffness component of about 22%, but they nevertheless fall within the large uncertainties associated with the experimental data reported and discussed by Feldman (1976 ▸). In addition, we are aware that the *C*_12_ stiffness component is positive in our work and negative in the cited study, a discrepancy caused by the different orientation of the Cartesian axes relative to the unit cell of the mineral (see above).

### 3.3. Electronic properties

**Figure 2:**
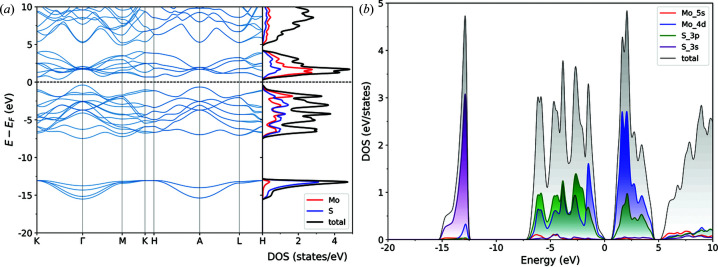
(*a*) Band structure and density of states (total and projected on the Mo and S atoms) of bulk molybdenite MoS_2_-2H. (*b*) Orbital-level details of the density of states, showing the contributions of the 3*s* (purple line) and 3*p* (green line) states of sulfur and of the 5*s* (red line) and 4*d* (blue line) states of molybdenum to the total density of states (DOS, black line).

**Figure 3:**
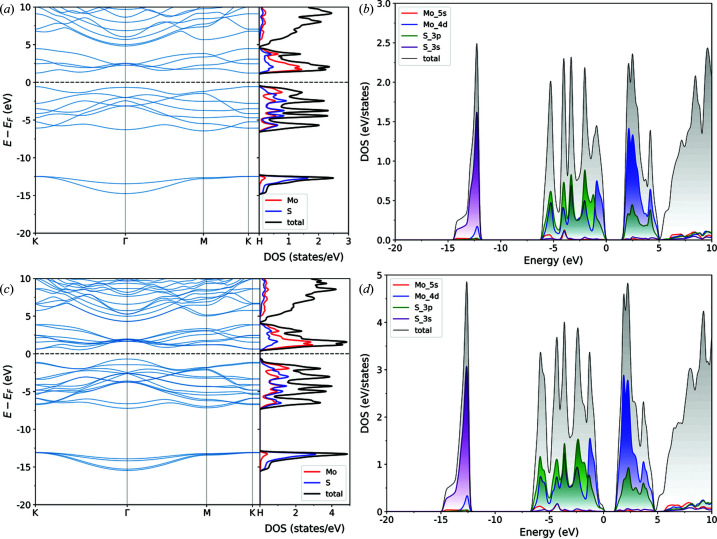
(*a*), (*c*) Band structures and densities of states (total and projected on the Mo and S atoms) of molybdenite (*a*) monolayer MoS_2_-1L and (*c*) bilayer MoS_2_-2L. (*b*), (*d*) Orbital-level details of the density of states, showing the contributions of the 3*s* (purple line) and 3*p* (green line) states of sulfur and of the 5*s* (red line) and 4*d* (blue line) states of molybdenum to the total density of states (DOS, black line) of (*b*) monolayer MoS_2_-1L and (*d*) bilayer MoS_2_-2L.

The electronic band structure and density of states for molybdenite bulk (MoS_2_-2H), calculated along the *K*–*G*–*M*–*K*–*H*–*L*–*A*–*H* path in the first Brillouin zone, are reported in Fig. 2 ▸, and the same results are presented in Fig. 3 ▸ for the MoS_2_-1L (monolayer) and MoS_2_-2L (bilayer) structures. It is possible to note from these results that there are four groups of bands in the calculated structure and density of states. The first group is related to valence bands located at low energy, between −12 and −15 eV, related mainly to the 3*s* orbital of the sulfur atom. The second group of valence bands, below the Fermi energy and separated from the first one by a large gap of about 7 eV, is the result of a visible hybridization of the 3*p* and 4*d* orbitals of S and Mo, respectively, with a small contribution from the 5*s* orbital of molybdenum around −5 eV. The third group is above the Fermi energy, representing the first range of conduction bands of molybdenite that are predominantly the result of the 4*d* orbitals of Mo and, to lesser extent, the 3*p* orbitals of S. The last group of (conduction) bands is located above *ca* 5 eV, given by a mixed contribution (hybridization) of the 5*s* and 5*d* states of molybdenum and the 3*p* orbitals of sulfur. Since the *d* orbitals are the main contributor to the bands located near the band gap, the bands are almost flat.

While these features are shared between the different structures (bulk and monolayer), there are important differences arising from the thickness of the materials. For instance, in the bulk MoS_2_-2H, the maximum energy of the highest occupied valence band is located on the Γ point and the minimum energy of the lowest occupied conduction band is on a point in the *K* → Γ path. This means that bulk molybdenite is a semiconductor with an indirect band gap of 0.79 eV. The band gap becomes a wider direct one, *E*_g_ = 1.90 eV, located on the *K* point for a monolayer of the mineral. However, even the bilayer MoS_2_ structure reverts the band gap from the direct *K*–*K*′ to the indirect one observed for the bulk mineral, albeit the gap is slightly higher (1.22 eV).

The present results are in very good agreement with previous theoretical ones reported in the literature. For example, Kumar & Ahluwalia (2012 ▸), using Troullier–Martin norm-conserving pseudopotentials as coded in the *SIESTA* software, calculated a band gap for bulk molybdenite of 0.75 eV by means of the local density approximation (LDA) functional, which increased to 1.05 eV when the authors employed the DFT functional of Perdew *et al.* (1996 ▸). For the MoS_2_-1L monolayer the obtained values were higher, namely 1.89 and 1.55 eV at the LDA and PBE levels of theory, respectively. Ataca & Ciraci (2011 ▸) performed similar simulations by means of the projector augmented-wave basis set, calculating the band gap of bulk MoS_2_ using LDA (or the generalized gradient approximation) as 0.72 eV (0.85 eV), whereas they obtained 1.87 eV (1.58 eV) for the molybdenite monolayer.

**Figure 4:**
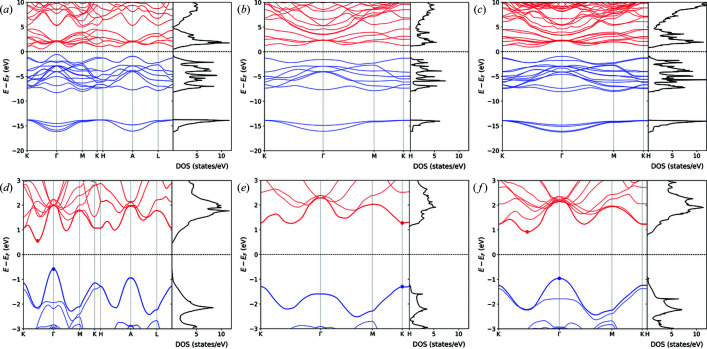
Band structures of (*a*) bulk MoS_2_-2H, (*b*) monolayer MoS_2_-1L and (*c*) bilayer MoS_2_-2L calculated at the GW level. Details of the band gap region are reported for the three structures in panels (*d*), (*e*) and (*f*), respectively. The blue and red dots in the highlighted regions are the maxima of the valence and minima of the conduction bands, respectively.

However, as also reported by Kumar & Ahluwalia (2012 ▸), all theoretical simulations on bulk MoS_2_-2H underestimated the band gap, as the experimental investigation by photocurrent spectroscopy assessed this value at 1.23 eV (Kam & Parkinson, 1982 ▸). This is a common issue related to both LDA and GGA (generalized gradient approximation) DFT functionals. To overcome this drawback, in the present work the GW approximation was employed, which allows us to describe the quasiparticle electronic properties. Fig. 4 ▸ shows the band structures and densities of state for the MoS_2_-2H bulk [Figs. 4 ▸(*a*) and 4 ▸(*d*)], the MoS_2_-1L monolayer [Figs. 4 ▸(*b*) and 4 ▸(*e*)] and the MoS_2_-2L bilayer [Figs. 4 ▸(*c*) and 4 ▸(*f*)]. The calculated band gaps are 1.14 eV (indirect), 2.57 eV (direct) and 1.88 eV (indirect) for the bulk mineral, monolayer and bilayer MoS_2_, respectively. Hence, the GW approach provides a better description of the band gaps of the MoS_2_-2H bulk and the layered structures. The results for the monolayer are also in very good agreement with the theoretical results of Zibouche *et al.* (2021 ▸), who calculated the band gap (2.68 eV) using the Sternheimer equation (Umari *et al.*, 2009 ▸).

### 3.4. Optical response function

The optical properties are due to the electronic transition from the occupied to the unoccupied state, in other words they are two-particle excitations. At the DFT level, the key quantity that is calculated is the frequency-dependent complex dielectric function ɛ(ω) = ɛ_1_(ω) + *iɛ*_2_(ω), with ɛ_1_ and ɛ_2_ the real and imaginary parts, respectively. This knowledge is very useful because the real component of ɛ(ω) provides information on the transmission of electromagnetic waves through the medium, whereas the ɛ_2_(ω) component is related to interband electronic transitions, *i.e.* single-particle excitations (Raether, 1980 ▸). The imaginary part of ɛ is calculated as a summation over empty states, according to the equations reported by Gajdoš *et al.* (2006 ▸), whereas ɛ_1_(ω) was obtained using the Kramers–Kronig relation. In addition, the dielectric function can also be calculated with the electric vector oscillating either parallel or perpendicular to the *c* axis, because each considered phase belongs to the hexagonal system. This is calculated with the following formulae:

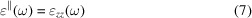

and

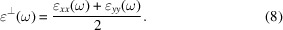




Other properties of interest can be derived from the complex dielectric function, such as the refractive index *n*(ω), the extinction coefficient κ(ω), the absorption coefficient α(ω), the energy-loss function EELS(ω) and the reflectivity *R*(ω), according to

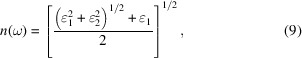




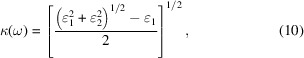




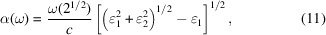




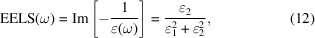




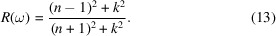

**Figure 5:**
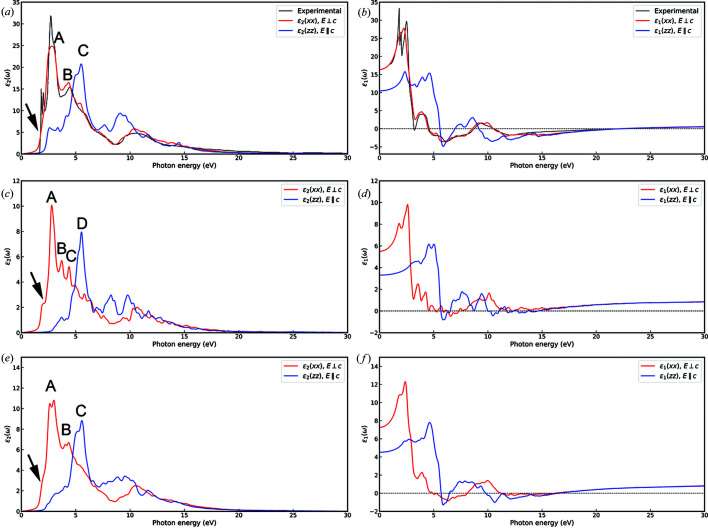
Imaginary (ɛ_2_) and real (ɛ_1_) components of the complex dielectric function, as obtained from the theoretical simulations at the PBE-D3 level for (*a*), (*b*) MoS_2_-2H, (*c*), (*d*) MoS_2_-1L and (*e*), (*f*) MoS_2_-2L. Red and blue lines refer to the on-plane (*xx*, **E** 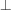 **c**) and out-of-plane (*xx*, **E** || **c**) electric field oscillations, respectively. Experimental data from Beal & Hughes (1979 ▸) are shown for direct comparison.

These optical properties were evaluated for each molybdenite model for the MoS_2_-2H, MoS_2_-1L and MoS_2_-2L structures, considering a high number of unoccupied bands to increase the accuracy of the results. Fig. 5 ▸ reports the dielectric function calculated in the photon energy range 0–30 eV, subdivided into real (ɛ_1_) and imaginary (ɛ_2_) parts. Bulk molybdenite [Figs. 5 ▸(*a*) and 5 ▸(*b*)] shows excellent agreement with the data measured by Beal & Hughes (1979 ▸) from the reflectivity spectra of MoS_2_-2H. As a consequence of the experimental setup, the comparison basis is the dielectric function calculated for the electric field oscillating perpendicular to the (001) plane. In this case, the calculated optical properties were not shifted to give a better match to the experimental findings, as was done in previous work (Kumar & Ahluwalia, 2012 ▸).

Three main peaks can be observed in the low-energy region (1–5 eV) of the imaginary part ɛ_2_, labelled A (2.76 eV), B (4.21 eV) and C (5.31 eV) according to the nomenclature proposed by Kumar & Ahluwalia (2012 ▸). The A peak corresponds to the C exciton (Funke *et al.*, 2016 ▸). There is good agreement with the theoretical data reported by Kumar and Ahluwalia, who calculated A = 2.9 eV, B = 4.5 eV and C = 5.1 eV, but our computational setting allows us to detect the exciton at 1.72 eV, corresponding to the *K*–*K*′ transition from the topmost valence (*v*1) band to the first conduction band (*c*1), marked with a black arrow in Fig. 5 ▸(*a*). However, the intensity of the signal is lower than the experimental one, and the second expected excitonic transition *c*2–*v*1 is not visible because of the overlap with the A peak. The present data are also in line with thermal ellipsometry data at 35 K measured by Le *et al.* (2019 ▸), who measured A = 2.91 eV and the *K*–*K*′ transition at 1.96 eV.

MoS_2_-1L presents four peaks in the imaginary part of the complex dielectric function, labelled A (2.82 eV), B (3.70 eV), C (4.39 eV) and D (5.54 eV) in Fig. 5 ▸(*c*), and they are in agreement with the results of Kumar & Ahluwalia (2012 ▸), *i.e.* A = 2.9 eV, B = 3.8 eV, C = 4.5 eV and D = 5.5 eV. In this case, the B peak is a new feature that appears because of the single-layer structure, whereas the other signals (A, C and D) are slightly shifted at higher energy with respect to the A, B and C peaks of the mineral bulk. A single excitonic *K*–*K*′ transition is also clearly visible at about 1.94 eV, in excellent agreement with that found at 1.9 eV by Funke *et al.* (2016 ▸), which was derived from spectroscopic ellipsometry measurements. A second peak at 2.05 eV was also measured by the same authors, which is related to spin–orbit coupling, *i.e.* a splitting of the topmost valence band in the single-layered MoS_2_ unit at about 140–150 meV. An example of this effect on the bands of 2D molybdenite can be seen in the experimental and theoretical work of Song *et al.* (2019 ▸) and Moynihan *et al.* (2020 ▸). Since spin–orbit coupling was not included in the present simulations, this second peak (*K*–*K*′ transition) is not visible in the dielectric function. Funke *et al.* (2016 ▸) found at about 3 eV the excitonic transition here labelled as A, in line with our calculations.

When the molybdenite structure is made of two layers, the shape of the dielectric function reverts to a form similar to that of bulk MoS_2_-2H [see Fig. 5 ▸(*e*)], presenting only three major peaks, A (2.73 eV), B (4.32 eV) and C (5.36 eV), in the range 0–5 eV.

In general, all the observed optical transitions occur in an energy range corresponding to electronic transitions between the *p* valence (occupied) bands of sulfur and the *d* conduction (unoccupied) bands of molybdenum (see Fig. 3 ▸), as also observed in previous work (Beal & Hughes, 1979 ▸; Kumar & Ahluwalia, 2012 ▸). According to the literature, the A peak in Figs. 5 ▸(*a*), 5 ▸(*c*) and 5 ▸(*d*) is mainly due to nearly parallel bands in the *M* → Γ path, *i.e.* band nesting. The low-energy excitons are instead related to electronic transitions from the Mo *d_z_* orbital, which is split at the *K* point in the Brillouin zone because of spin–orbit interaction.

The real part of the dielectric function of the MoS_2_-2H structure is also in very good agreement with the experimental findings (Beal & Hughes, 1979 ▸; Funke *et al.*, 2016 ▸), with values at zero energy of 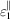 = 10.42 eV and 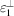 = 16.43 eV that are in line with those measured from experiments, *e.g.* 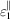 = 10.42 eV and 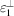 = 16.8 eV (Beal & Hughes, 1979 ▸). These values are reduced when going from bulk to the monolayer ( 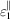 = 3.29 eV and 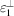 = 5.49 eV) and the bilayer ( 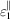 = 4.53 eV and 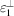 = 7.25 eV), showing a trend that increases with the number of MoS_2_ units in the structure. Our results are also in line with the theoretical simulations of Kumar & Ahluwalia (2012 ▸), who calculated 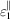 = 8.9 eV and 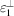 = 12.8 eV for the MoS2-2H bulk, and 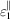 = 3.0 eV and 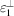 = 4.8 eV for the molybdenite monolayer. A better agreement can be observed by comparing our results with the theoretical ones of Ben Amara *et al.* (2016 ▸), who obtained 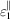 = 8.3 eV and 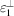 = 15.4 eV from PBE simulations.

**Figure 6:**
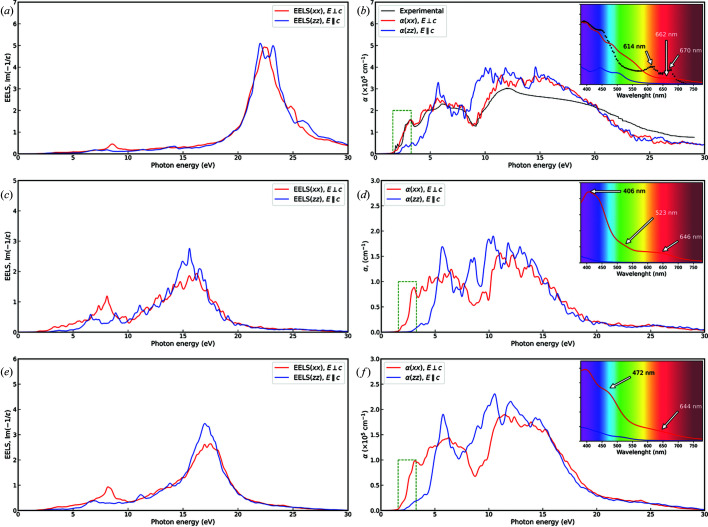
Calculated EELS and optical absorption α for (*a*), (*b*) MoS_2_-2H, (*c*), (*d*) MoS_2_-1L and (*e*), (*f*) MoS_2_-2L (ɛ_2_). Red and blue lines refer to the on-plane (*xx*, **E** 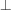 **c**) and out-of-plane (*xx*, **E** || **c**) electric field oscillations, respectively. Experimental data from Beal & Hughes (1979 ▸) are shown for direct comparison. The green dashed boxes in panels (*b*), (*d*) and (*f*) show the part of the absorption spectrum in the visible region, which is reported in the upper right inset.

The calculated electron energy-loss spectroscopy (EELS) spectra (Fig. 6 ▸) provide further information on the plasmon modes, *i.e.* the collective oscillation of valence or conduction electrons in a material. These modes are related to transitions from the π and σ bonding states to the respective antibonding states π* and σ*, which occur in the EELS spectra as low-energy π and high-energy π+σ plasmons (interband plasmons). Such plasmon modes are present in the EELS spectrum when the real part of the dielectric function ɛ_1_(ω) crosses zero with a positive slope. In bulk molybdenite (MoS_2_-2H), the π plasmon falls at 8.52 eV, whereas the π+σ one can be seen at 22.55 eV for **E** 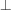 **c**. For an electric field oscillating parallel to the *c* axis, the π+σ plasmon presents two peaks at 22.98 and 23.17 eV, and no other well defined signals are visible in the spectrum. A similar trend is observed for layered MoS_2_, but both the monolayer and bilayer systems show a red shift of these plasmon signals (for **E** 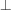 **c**), at 8.06 eV (π) and 16.17 eV (π+σ) for MoS_2_-1L, and 8.31 eV (π) and 17.44 eV (π+σ) for the MoS_2_-2L model. For the electric field parallel to the *c* axis, the π+σ plasmon falls at 15.55 and 17.01 eV for MoS_2_-1L and MoS_2_-2L, respectively. These theoretical results are in very good agreement with the experimental EELS measurements conducted via scanning transmission electron microscopy (STEM) by Moynihan *et al.* (2020 ▸), who obtained for MoS_2_-2H 8.6 and 23 eV for the π and π+σ plasmons, respectively. Compared with the previous DFT simulations at the PBE level (Kumar & Ahluwalia, 2012 ▸), our results are in general agreement, with the π plasmon falling at lower energies than previously reported, *i.e.* 9.2 and 8.6 eV for the bulk and monolayer structures, respectively.

The optical absorption α is also reported in Fig. 6 ▸, in the photon energy range 0–30 eV. The simulation results for bulk molybdenite [Fig. 6 ▸(*b*)] are in excellent agreement with the experimental ones of Beal & Hughes (1979 ▸) up to about 15 eV, and then there is some deviation between the curves due to the data extrapolation performed by those authors. In the region of the visible spectrum [Vis, 380–780 nm, see the inset in Fig. 6 ▸(*b*)], MoS_2_-2H strongly absorbs violet light, with two peaks between 400 and 450 nm. Experimentally, two absorption peaks were found at 614 and 670 nm related to the *K*–*K*′ transitions, whereas a single almost flat signal centred at 662 nm was obtained from the simulations. Similar general figures were found for the MoS_2_-1L [Fig. 6 ▸(*d*)] and MoS_2_-2L [Fig. 6 ▸(*f*)] systems, with absorption peaks in the red (*ca* 650 nm) and violet regions (about 400 nm) of the visible spectrum.

### 3.5. Phonon properties

Bulk molybdenite (space group *P*6_3_/*mmc*, point group 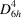 ) has six atoms in the unit cell, resulting in 18 degrees of freedom that, in the Γ point and from group-theory analysis, have the following irreducible representations (irreps):

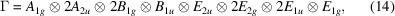

with Γ_a_ = 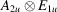 and Γ_o_ = 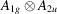 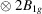 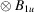 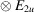 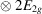 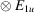 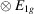 being the acoustic and optical modes, respectively. Modes with character *A* or *B* are non-degenerate, whereas the *E* modes are doubly degenerate. Vibrational motions that are symmetric (*gerade*, *g*) and anti-symmetric (*ungerade*, *u*) with respect to the inversion centre of the crystal are active in Raman and infrared spectroscopies, respectively.

The single layer of MoS_2_ ( 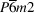 space group, *D*_3*h*_ point group) has only nine degrees of freedom and different zone-centre irreps,

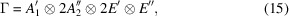

with Γ_a_ = 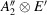 and Γ_o_ = 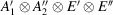 . If we consider a correlation with the *D*_6*h*_ point group, it is possible to associate the *E*′′ and 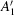 modes of the monolayer with the *E*_1*g*_ and *A*_1*g*_ modes of the bulk, respectively. Instead, while the bilayer MoS_2_-2L has the same degrees of freedom as the mineral bulk, the 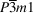 space group ( 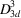 point group) leads to different irreps,

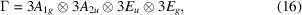

where Γ_a_ = 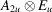 and Γ_o_ = 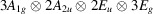 . Again, *gerade* (*A*_1*g*_ and *E*_*g*_) and *ungerade* modes (*A*_2*u*_ and *E*_*u*_) are active in Raman and IR, respectively.

**Figure 7:**
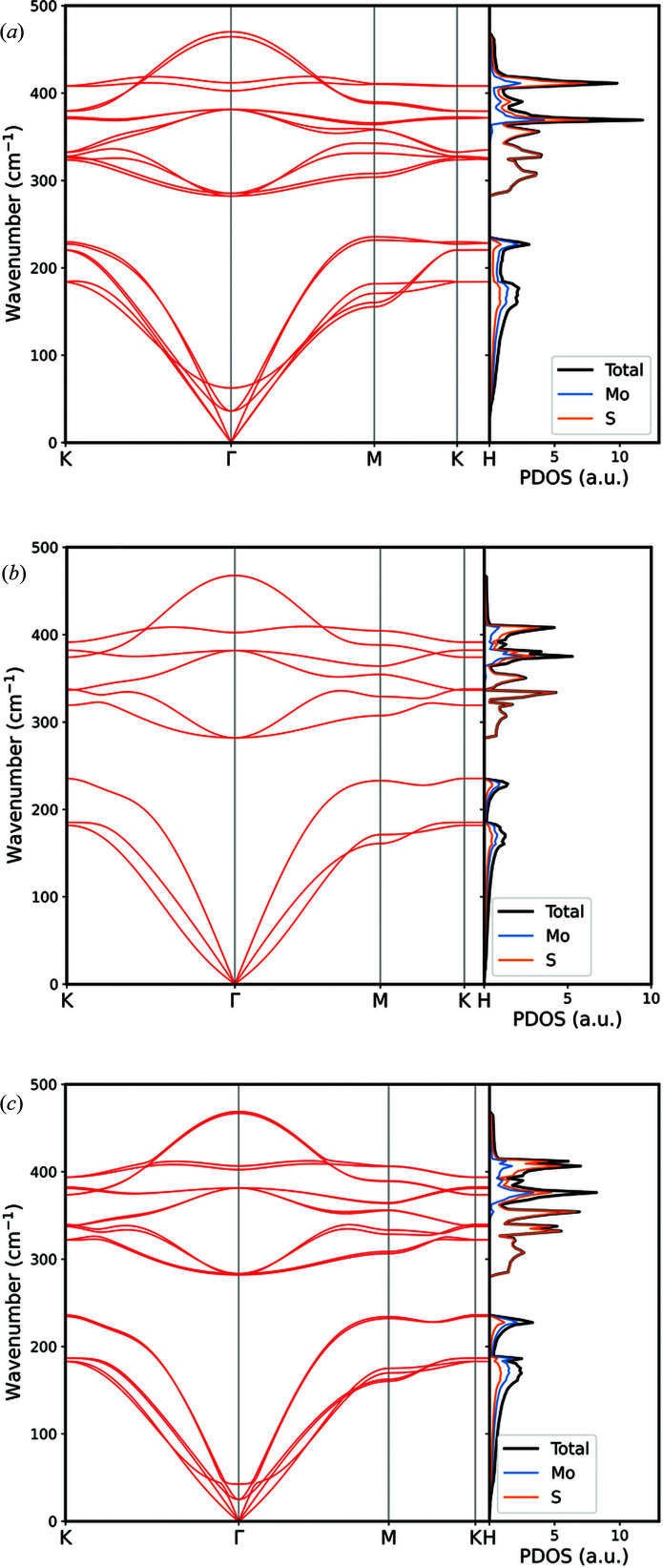
Phonon band structures and atom-projected phonon densities of states for (*a*) bulk MoS_2_-2H, (*b*) single-layer MoS_2_-1L and (*c*) bilayer MoS_2_-2L structures.

Γ-point frequencies are reported in Table 3 ▸, alongside selected results from both experiments (Funke *et al.*, 2016 ▸; Wieting & Verble, 1971 ▸) and theoretical simulations (Ataca *et al.*, 2011 ▸; Jiménez Sandoval *et al.*, 1991 ▸). The phonon dispersion curves for each model along the *K*–Γ–*M*–*K*–*H* path in the first Brillouin zone are shown in Fig. 7 ▸. No negative value of the phonon branch was found, meaning that the bulk and the free-standing layered structures are stable. In general, there is very good agreement between the different results, with mean absolute differences less than 3 cm^−1^. Two Raman modes are quite sensitive to the number of layers in the structure, *i.e.* the No. 7 *E*_2*g*_ and No. 10 *A*_1*g*_ modes in MoS_2_-2H, the former decreasing and the latter increasing with the number of layers of the 2D material, in agreement with previous experimental observations (Funke *et al.*, 2016 ▸; Lee *et al.*, 2010 ▸). This is an anomalous behaviour that deviates from the classical model of coupled harmonic oscillators, where it is expected that the cited *E*_2*g*_ and *A*_1*g*_ modes should both stiffen on increasing the thickness of the molybdenite. This is due to the stacking of the MoS_2_ layers along the *c* axis, which affects the intralayer bonding (see Table 1 ▸) and hence the force constants of the vibrational motion of the atoms. Interestingly, according to Wieting & Verble (1971 ▸), the frequency difference between the *E*_1*u*_ and *E*_2*g*_ modes in the bulk is associated with the interlayer interaction, which is quite low in our simulations (no more than 3 cm^−1^). This extends to the bilayer model MoS_2_-2L, considering the *E*_*u*_ and *E*_*g*_ vibrations. However, while the absolute frequency shift of the *A*_1*g*_ → 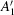 mode is quite in line with the experimental one (*ca* 4 cm^−1^) of Lee *et al.* (2010 ▸), the *E*_2*g*_ → *E*′ mode is much less affected by the thickness of the material, in agreement with previous theoretical observations (Ataca *et al.*, 2011 ▸). We suppose that this is due to the absence of any interaction of the MoS_2_ monolayer or bilayer with the support (*e.g.* SiO_2_ or sapphire) that is commonly employed in experimental measurements.

Regarding the phonon dispersion (Fig. 7 ▸), there is a satisfactory agreement with previous inelastic neutron scattering data measured by Wakabayashi *et al.* (1975 ▸) along the Γ–*M* path ([100] direction) for MoS_2_-2H. No significant differences in the phonon density of states were observed in our simulations by changing the number of layers in the system.

Quite good agreement was found by comparing the present results with those of Ataca *et al.* (2011 ▸), who performed PW91-D simulations for the bulk and monolayer of molybdenite for both the phonon branches and the density of states. Their GGA+D approach resulted in *E*_1*g*_ = 286.6 cm^−1^, *E*_2*g*_ = 378.5 cm^−1^ and *A*_1*g*_ = 400.2 cm^−1^ for MoS_2_-2H, and *E*′ = 380.2 cm^−1^ and 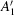 = 406.1 cm^−1^ for MoS_2_-1L. It is worth noting the opposite behaviour of the *A*_1*g*_ → 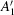 frequency shift, which is explained by the different unit-cell parameters between theory and experiment.

## 4. Conclusions

In this work, with the density functional theory method, using plane-wave basis sets, the PBE functional and the DFT-D3 scheme to include van der Waals interactions, we have investigated different properties of bulk molybdenite MoS_2_-2H and its monolayer (MoS_2_-1L) and bilayer (MoS_2_-2L) structures. The scope was to provide a detailed cross-correlated analysis of these materials on the atomic scale, both to increase the knowledge of them and as a comparison basis for future work.

We have noted subtle variations in the crystal chemistry of MoS_2_ when simulated in layers, in particular the bond distances and angles, with binding energy values in agreement with experimental measurements that employed the peeling-to-fracture method. The selected PBE-D3 approach also described the structure of bulk MoS_2_-2H, with the expected unit-cell volume at 0 K smaller than that from room-temperature XRD refinements. The stiffness of bulk MoS_2_-2H was also in line with the only experimental work reported in the literature, with a slight overestimation of the *C*_33_ elastic modulus and an underestimation of the *C*_13_ one as a result of the approximations introduced in the simulations, chiefly the lack of thermal effects (athermal results at 0 K without any zero-point contribution).

The band gaps of bulk molybdenite, MoS_2_-1L and MoS_2_-2L calculated at the GGA (GW) level were an indirect 0.79 eV (1.14 eV), a direct 1.90 eV (2.57 eV) and an indirect 1.22 eV (1.88 eV), respectively, in line with both experimental data and theoretical simulations that considered quasi-particle excitations. We observed from the atom-projected density of states that the 3*p* orbitals of S hybridize with the 4*d* ones of Mo at the top of the valence band and at the bottom of the conduction band, whereas the core states are due to the 3*s* orbitals of the sulfide ions. We expect that hybrid functionals, such as HSE06, could perform similarly but with much higher computational costs, especially when using plane-wave basis sets.

The calculated complex dielectric function showed the direct (zero-momentum **q**) electronic transitions (imaginary part) and the plasmonic resonances (EELS spectrum) in the photon energy range 0–30 eV, which agree with recent experimental measurements. Different absorption peaks in the visible portion of the light spectrum were observed according to the number of layers of molybdenite.

Finally, we studied the IR and Raman vibrations at the zone centre and the phonon dispersion relations of the three MoS_2_ models (bulk, monolayer and bilayer). Our simulations confirm the experimental evidence that the No. 7 *E*_2*g*_ mode decreases and the No. 10 *A*_1*g*_ mode increases with the number of layers of the bidimensional material. However, the extent of the former vibration is less than expected because in our setting the mono- and bilayer are simulated in a vacuum, without any interaction with typical substrates used in experimental work, such as silica or sapphire, that could affect the force constant of these modes.

## Figures and Tables

**Table 1:** Calculated lattice parameters *a* and *c* (Å), S—S and Mo—S bond distances (Å), S—Mo—S bond angle *Θ* (°), interlayer distance *d*_I_ (Å), internal parameter *z* (dimensionless), bulk modulus (*K*_0_, GPa), interlayer binding energy Δ*E*_bind_ (meV per atom) and cohesive energy Δ*E*_coh_ (eV per atom) for bulk (MoS_2_-2H) and layered (MoS_2_-1L and MoS_2_-2L) structures, compared with previous theoretical and experimental (Exp.) results The asterisk (*) marks fixed parameters in the present theoretical simulations.

System	Method	*a*	*c*	S—S	Mo—S	Θ	*d*_I_	*z*	*K*_0_	Δ*E*_bind_	Δ*E*_coh_
Bulk (MoS_2_-2H)	PBE-D3^*a*^	3.149	12.080	3.130	2.399	81.46	2.910	0.130	42 (2)	47	5.438
	LDA^*b*^	3.125	12.137	3.12	2.39					55	
	PW91^*b*^	3.215	15.540							3	
	PW91-D2^*b*^	3.220	12.411	3.150	2.436	80.56			44	53	5.105
	PBE^*c*^	3.18	14.68					0.143	2		5.12
	PBE-D2^*c*^	3.19	12.42					0.125	39		5.37
	vdW-DF^*d*^	3.23	12.6						39	60	
	Exp.	3.160^*e*^	12.294^*e*^	3.190^*e*^	2.410^*e*^			0.121^*e*^	53.4^*f*^		5.18^*g*^
											
Monolayer (MoS_2_-1L)	PBE-D3^*a*^	3.149	20*	3.137	2.401	81.58		0.078			5.344
	LDA^*h*^	3.11		3.11	2.37	81.62					6.35
	PW91^*b*^	3.20		3.13	2.42	80.69					5.18
	PW91-D2^*b*^	3.220		3.153	2.437	80.62					5.052
	Exp.	3.20^*i*^									
											
Bilayer (MoS_2_-2L)	PBE-D3^*a*^	3.149	30*	3.134	2.400	81.53	2.929	0.052		22	5.389
										

References: (*a*) present work, (*b*) Ataca *et al.* (2011 ▸), (*c*) Bučko *et al.* (2010 ▸), (*d*) Rydberg *et al.* (2003 ▸), (*e*) Bronsema *et al.* (1986 ▸), (*f*) Aksoy *et al.* (2006 ▸), (*g*) Raybaud *et al.* (1997 ▸), (*h*) Ataca & Ciraci (2011 ▸) and (*i*) Joensen *et al.* (1986 ▸).

**Table 2:** Elastic moduli of MoS_2_-2H polytype (in GPa), as obtained from different theoretical and experimental (Exp.) approaches

	PBE-D3^*a*^	Exp.^*b*^	HSE06-D2^*c*^	PBE^*d*^	Hartree–Fock^*e*^
*C*_11_	231.82	238	238	211	255
*C*_12_	55.20	−54	64	49	−38
*C*_13_	10.63	23	12	3	17
*C*_33_	63.47	52	57	37	35
*C*_44_	21.35	19	18	30	15

References: (*a*) present work, (*b*) Feldman (1976 ▸), (*c*) Peelaers & Van de Walle (2014 ▸), (*d*) Wei *et al.* (2010 ▸) and (*e*) Alexiev *et al.* (2000 ▸).

**Table 3:** Zone centre vibrational frequencies ν (cm^−1^) of the bulk (MoS_2_-2H), monolayer (MoS_2_-1L) and bilayer (MoS_2_-2L) models, as obtained from PBE-D3 simulations, compared with experimental results (Exp.) where these are available Activity in infrared and/or Raman spectroscopy is indicated with IR or R, respectively.

System	Mode	Irrep^*a*^	ν^*a*^	IR/R	Exp.^*b*^	Exp.^*c*^	VFF^*d*^	PW91+D^*e*^
MoS_2_-2H	1	*E*_1*u*_	0.0					
	2	*A*_2*u*_	0.0					
	3	*E*_2*g*_	36.0	R		32		
	4	*B*_1*g*_	62.4	R				
	5	*E*_2*u*_	282.1	IR				285.0
	6	*E*_1*g*_	285.3	R		287		286.6
	7	*E*_2*g*_	381.0	R	383.5	383		378.5
	8	*E*_1*u*_	381.3	IR				378.8
	9	*B*_2*u*_	402.5	IR				395.4
	10	*A*_1*g*_	411.7	R	408.9	409		400.2
	11	*A*_2*u*_	464.5	IR				456.9
	12	*B*_1*g*_	470.1	R				460.3
								
MoS_2_-1L	1	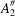	0.0					
	2	*E*′	0.0					
	3	*E*′′	281.9	R			280	287.1
	4	*E*′	381.8	IR, R	384.4		384	380.2
	5	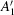	402.3	R	403.1		407	406.1
	6	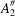	467.7	IR			481	465.0
								
MoS_2_-2L	1	*E*_*u*_	0.0					
	2	*A*_2*u*_	0.0					
	3	*E*_*g*_	24.8	R				
	4	*A*_1*g*_	42.6	R				
	5	*E*_*u*_	281.9	IR				
	6	*E*_*g*_	283.5	R				
	7	*E*_*g*_	381.3	R	383.9			
	8	*E*_*u*_	381.4	IR				
	9	*A*_2*u*_	402.3	IR				
	10	*A*_1*g*_	406.5	R	404.6			
	11	*A*_2*u*_	466.8	IR				
	12	*A*_1*g*_	468.8	R				

References: (*a*) present work, (*b*) Funke *et al.* (2016 ▸), (*c*) Wieting & Verble (1971 ▸), (*d*) Jiménez Sandoval *et al.* (1991 ▸) using the valence force field (VFF) model and (*e*) Ataca *et al.* (2011 ▸) using the DFT/PW91-D approach.
